# Decision-making regarding immediate vs. interval postpartum levonorgestrel intrauterine device insertion timing

**DOI:** 10.1186/s40834-023-00221-3

**Published:** 2023-03-20

**Authors:** Abigail Davenport, Laura Morello, Kavita Shah Arora

**Affiliations:** 1 Section of Female Pelvic Medicine and Reconstructive Surgery, MedStar Washington Hospital Center, Washington, DC United States of America; 2 Department of Bioethics, Case Western Reserve University, Cleveland, OH United States of America; 3 Department of Obstetrics and Gynecology, University of North Carolina at Chapel Hill, Chapel Hill, NC United States of America

**Keywords:** Postpartum, Contraception, Decision-making, Intrauterine device, Qualitative

## Abstract

**Background:**

Understanding decision-making for contraception initiation timing postpartum may help guide patients in selecting a contraceptive method most aligned with their reproductive goals. The objective of this study was to explore the decision-making process in patients who chose immediate postplacental (IPP) levonorgestrel intrauterine device (LNG IUD) insertion versus interval insertion at the postpartum follow-up visit.

**Methods:**

We recruited English-speaking, reproductive-aged adult postpartum participants who received either an IPP or interval postpartum LNG IUD from September to December 2017 at MetroHealth Medical Center. We conducted interviews over the phone utilizing a pilot-tested, semi-structured interview guide. Interview topics included past experiences with contraception, provider counseling, intrapartum factors, and current experiences after IUD insertion.

**Results:**

We interviewed 20 participants (12 IPP and eight interval IUD recipients). Participants receiving an IPP IUD described convenience, desire for immediate contraception, pain control and availability of alternative contraception options as influential for their decision. Patients who received an interval IUD performed outside research, focused on the events surrounding delivery, and generally favored additional recovery time before obtaining an IUD. Patients who received interval IUDs were often not aware that IPP IUDs were available. Early, frequent, and comprehensive counseling was viewed favorably when compared to counseling upon arrival to the laboring unit. While overall there was congruence of participant expectations and experiences, unexpected expulsion affected desire for future IUD use in some participants.

**Conclusion:**

Providers should be mindful that prior experience and knowledge as well as delivery room considerations affect insertion timing decision-making.

**Trial registration:**

N/A

## Introduction

Long-acting reversible contraception (LARC) includes intrauterine devices (IUDs) and the etonorgestrel rod. It has several advantages over other contraceptive alternatives including increased efficacy, continuation rates, and safety profiles [1, 2]. In recent years, LARC has become available in the immediate postpartum period. For the provider, immediate postpartum (IPP) LARC is attractive as it has the potential to decrease short-interval pregnancy, ensure reliable contraception for patients who may not attend their postpartum visit, and is cost-effective [3]. However, understanding the manner in which patients’ reproductive goals influence placement timing postpartum is equally essential when providing comprehensive contraception counseling.

Several studies have explored the experiences of patients who received postpartum LARC [4–6]. In one study, barriers to obtaining contraception remote from delivery and convenience influenced patients obtaining IPP IUD insertion [4]. Additionally, improved antenatal counseling was associated with increased use of IPP IUDs, and patients in one IPP LARC program felt that counseling prior to hospital admission was preferred to make a fully informed decision [5, 6]. While the experiences of patients for either IPP or interval postpartum IUD timing were described, the decision-making surrounding the choice between these two options is currently unknown, as noted in a recent literature review [7].

Our study aimed to explore the decision-making process surrounding IPP versus interval postpartum levonorgestrel IUD insertion timing using a qualitative approach.

## Methods

In this qualitative study, we conducted semi-structured open-ended interviews with postpartum patients at least 18-years-old who received either an IPP or interval postpartum levonorgestrel (LNG) IUD at MetroHealth Medical Center (MHMC) between September 2017 to December 2017. MHMC is a safety net hospital located in Cleveland, OH. We identified potential study candidates via our hospital’s linked electronic medical record-based billing reports and cross-checked via our pharmacy’s dispensing logs. For the purpose of this study, we defined an IPP IUD as one placed within ten minutes of completion of the third stage of labor. Obstetrics and gynecology and family medicine clinicians provide prenatal and postpartum care at MHMC and receive formal training in IPP IUD insertion. Inpatient copper IUD insertion and subcutaneous implant placement were not offered at MHMC during the study period. We defined an interval postpartum IUD as placement in the office between four and eight weeks following parturition. Content of contraception counseling is left to the discretion of the healthcare provider. We excluded non-English-speaking patients due to cost limitations and ease of conversation. Institutional review board (IRB17-00387) approval was obtained at MHMC.

We selected potential participants for interview once six months had elapsed since placement of their LNG IUD as this timepoint is congruent with existing studies examining postpartum IUD insertion and allows for patient reflection on their experiences while minimizing recall bias [7]. We contacted potential participants by telephone. Purposeful sampling was used in order to more evenly represent both groups of IUDs as more patients received an IPP IUD compared to an interval IUD. We attempted to match those receiving an interval IUD to those receiving an IPP IUD by month of insertion. We obtained verbal consent over the phone. Remuneration in the form of a $15 online gift card was offered to participants.

Basic demographic information including age, parity, ethnicity, and insurance type was collected. We used a pilot-tested interview guide including questions about participants’ reproductive goals, previous contraceptive experiences, social and medical influences, and current experiences with their LNG IUD to direct the conversation (Table 1).

**Table 1:** Selection of interview guide questions

1. Have you used birth control or contraception in the past? What kind?
2. What made you choose that form of birth control?
3. Describe the conversation you had with your provider about birth control during your prenatal visits.
4. Why did you decide you wanted an IUD after having your baby?
5. Describe why you wanted your IUD placed right after having your baby or at your postpartum visit.
6. What did your doctor tell you about the timing of placement? What effect did this discussion have on your decision to get the IUD?
7. What would you say were the most influential factors that made you decide to get your IUD in the delivery room versus in the office (or vice versa)?
8. Describe your experience of getting the IUD.
9. How do you feel about your IUD now?

We transcribed the audio-recorded interviews into text verbatim and uploaded all transcriptions into Dedoose—a qualitative analysis software program. We developed a codebook and dictionary containing both a priori codes, based on the interview guide categories, as well as emergent codes, using the transcripts of five initial interviews. We then coded the remainder of the interviews using a successive coding passes strategy, beginning with open coding of content at the level closest to the content of the text and continuing through broader and more analytic codes [8].

Two project staff (A.D. and L.M.) independently coded each transcript followed by a process of consensus coding. AD was a resident physician in OBGYN and LM is a project manager with expertise in qualitative research methodologies. After the completion of the 20 interviews, no new codes were generated and we reached thematic saturation. We examined the presence or absence of particular coding categories across the interviews to look for areas of discussion that were unique to particular groups.

## Results

**Fig 1.**
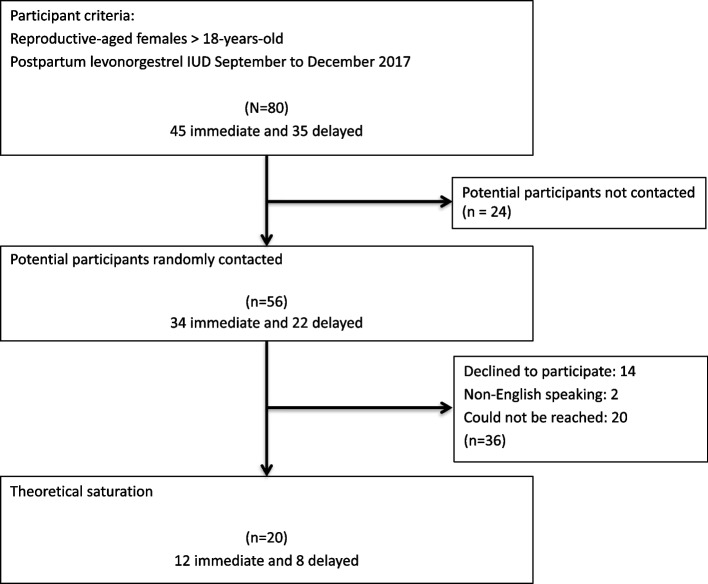
Participant selection flow diagram

Eighty patients received a postpartum LNG IUD (45 IPP and 35 interval) at our institution between September and December of 2017. Of these patients, we contacted a total of 56 (34 IPP and 22 interval) for study participation. Fourteen patients declined to participate and two were excluded due to language barriers. Twenty patients could not be contacted. Twenty participants were interviewed. Twelve had received an IPP IUD and eight received an interval IUD. Figure 1 describes the recruitment process.

While comparative statistics are not presented due to the small sample sizes inherent in qualitative research, those in the IPP IUD group tended to be Black, more parous, and have public insurance. Table 2 describes participant characteristics including age, ethnicity, parity, and insurance type data.

**Table 2:** Participant characteristics

Characteristic	Immediate *n*=12	Interval *n*=8
Age	29.8 (19-39)	31.8 (19-42)
Ethnicity		
Black	9 (0.75)	4 (0.5)
White	3 (0.25)	4 (0.5)
Parity	3 (1-8)	2 (1-4)
Insurance		
Public	11 (.92)	5 (0.625)
Private	1 (.08)	2 (0.25)
Uninsured	--	1 (0.125)

Data are median (interquartile range) or n (proportion).

**Fig 2.**
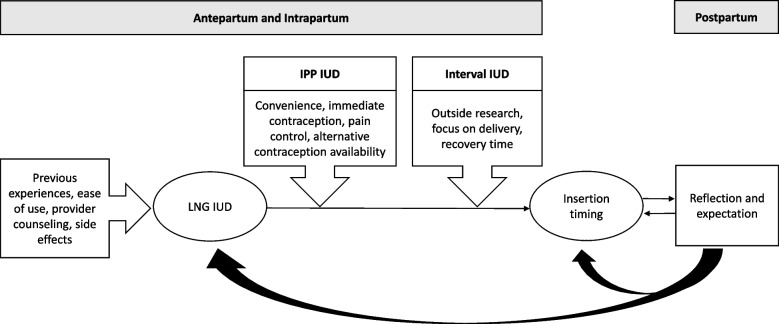
Schematic of immediate postplacental versus delayed insertion timing decision-making for the levonorgestrel intrauterine device

We present detailed descriptions of the categories and themes identified during our analysis below with a schematic depicted in Figure 2. Table 3 describes our coding framework with additional quotations.

**Table 3:** Summary of coding framework with additional quotations

**Category and Domains**	**QUOTE**
Convenience	“I was leaving the hospital on birth control. You know, cause stuff does happen. So I felt comfortable knowing that I was on a birth control leaving.”
Complication rates	“I’ve never had an IUD before, um, I was… I mean, I was a little nervous about getting it. So yeah, [I chose the delayed IUD] to lessen my chances of [expulsion].”
Pain	“When I had got it done like the first time (an in-office IUD). I was cramping. I really couldn’t stand up straight. And then I had to walk with a hunch because it was really hurting. But [the immediate IUD], it was easy.”
Knowledge	“I brought up the IUD when I got asked questions about it. [My doctor] explained to me more about it and he kinda reassured me. He recommended the [delayed IUD] instead of the [immediate] one. So yeah, it just kinda went from there, basically.”
Focus on delivery	“It was nice that [my doctor] brought [the IUD] up. Cause at the time it wasn’t something you were thinking about. I was thinking about the baby coming into the world.”
Recovery time	“I think it probably woulda been better if [the IUD] woulda got put in…. after the fact I had the baby, the swelling had went down, and you know, everything come back to where it should be. I shoulda got it then.”
Availability of alternatives	“I would be 100% for getting my tubes tied, but my OB can’t get to them. So my other option that is 99% effective, almost 100%, is getting an [immediate] IUD where I don’t have to worry about things.”
Reflection	“This one is a lot different and I feel a lot more positive about [the immediate IUD] and comfortable with it than the first one. So I’m so happy with it.”
Expectation	“It was a little different than what I expected. And it was a little scary [when it expulsed]…”

### Prior experience and knowledge

Participants’ prior experience with contraception and knowledge regarding IUDs impacted their decision-making. Almost all (19) participants had previously used contraception of any type. Methods requiring daily dosing or multiple clinic visits were viewed unfavorably. All participants who had previously used contraception described concerns about side effects.

Many participants described convenience as the most important factor influencing IPP insertion timing. “It was pretty easy. I was already technically able to go out and just heal and not have to worry about going to another doctor’s appointment and scheduling it,” said one participant. Several participants chose an IPP IUD because they wanted to leave the hospital with contraception and were concerned about short interval pregnancy: “My kids are really a couple months apart... So they’re really like back-to-back. So since I’d kinda just went through it, I was like, ‘Let’s just do [the IPP IUD]! I don’t want to get pregnant no more! I’m over it!’”

The influence of complication rates specific to the different IUD insertion timing options was highly individualized. The increased rate of IUD expulsion with IPP IUD insertion was perceived negatively by almost half of the study participants. “I’ve never had an IUD before… so [I chose the interval IUD] to lesson my chances of anything happening,” stated one participant who was concerned about the increased expulsion risk with IPP IUD. Others were less concerned: “If it falls out, that’s ok, I’ll just call and get an appointment scheduled [for it] to be placed back [in].” Several participants had not been counseled by their provider about expulsion: “I ain’t never heard about it falling out.” Additionally, half of the participants who received an interval IUD stated they would have chosen an IPP IUD if they had known the risk of perforation was higher in the office: “That woulda made a difference... that’s kinda scary.” Still, others were less worried: “It’s small statistics that it happens.”

Anesthesia was a strong consideration for many participants. “I told them to put it in while I was numb on the table and didn’t feel anything,” said one IPP IUD recipient. Several participants who previously received in-office IUDs described cramping and pain with placement and subsequently favored an IPP IUD under epidural anesthesia: “It was different the second time I got it. Like I said, it didn’t hurt like when I got the first one. I was shocked.” Another participant had a relatively painless experience with a previous IUD insertion in the office and subsequently elected to obtain an interval IUD: “I thought it was simple. I thought that when they put it in it was simple... I had very low cramping. It was fine.”

Knowledge regarding IUD insertion was influenced by independent research, conversations with family and friends, and provider counseling. Outside research (i.e. that occurring outside of the clinical encounter via the internet, television, pamphlets, etc.) was more commonly performed by interval IUD recipients. “I did a lot of online research so… I actually went to like the Planned Parenthood website and I went to the [IUD] website,” said one participant. Another participant read about interval insertion and elected to wait: “I feel like I’d read that you were supposed to wait to get it in. Like, it was better to do it after you’d had a cycle or something.”

Most participants described conversations with partners, friends, or family members about postpartum IUDs. “My husband had stopped that last minute... It was too permanent,” said one participant who obtained an IPP IUD rather than a tubal ligation. Another IPP IUD recipient had a similar conversation with one of her friends: “One of my best friends, she got her tubes tied. She got one child and she’s like 19, and she regrets getting her tubes tied cause she wants more kids.” Other participants were less influenced by the opinions of their friends: “Everyone had different point of views about it or opinions about it… But everyone’s body’s different so it just depends on what your body will take.”

Provider-driven contraception counseling varied in timing, frequency, and content. Participants described conversations preceding pregnancy, some as late as at the time of cesarean section, while others had not been counseled about a specific option at all. Most participants had decided on insertion timing during conversations with their provider prior to laboring. Only three participants were undecided about insertion timing on arrival to the laboring unit. One participant chose an interval insertion while the other two opted for IPP insertion.

In general, early and frequent counseling afforded participants time to weigh their options while delayed counseling about IUD insertion timing (i.e. on arrival to the laboring unit) was viewed as coercive. “[My doctor] has been talking to me about contraception for years,” said one participant. Another participant described continued IUD education antenatally and during labor: “Even if I didn’t get everything from [my doctor]… the nurse was always there. She was like, “What did you think about this? What did you think about that?” So it kinda went together... I never just heard something one time.” One IPP IUD recipient had not heard about this option until arrival to the laboring unit: “The first time telling me about it was when I was already in the room getting prepped to get cut... It [felt] like, not getting forced to do something, but like, doing something you didn’t… get a chance to really think about... It is important to me to think about the situation before I decide to do it.” Her IUD later expulsed postpartum and she chose to have it replaced.

None of the interval IUD recipients could recall being counseled about IPP insertion upon arrival to the laboring unit and also could not recall being counseled about this option by their antenatal provider. “If I would have known about [the IPP option], I would have gotten it a long time ago,” said one interval IUD recipient. “I probably would’ve done it,” said another interval IUD recipient, “Cause it would’ve been taken care of right then. I would have had it done right then.”

### Delivery room considerations

Considerations at the time of delivery (desire to focus on delivery, recovery time, and availability of other contraceptive options) influenced participants’ “readiness” to receive an IUD at the time of delivery or in the office. For some, the stresses of labor and imminent delivery caused them to favor interval IUD placement: “I felt so freaked out with so many other things that I don’t know if anyone tried to say, ‘Well, postpartum….’ I would have been like, ‘Are you kidding me? Can we just focus on getting this baby out.’”

Several participants favored interval IUD placement as they felt this gave their body adequate recovery time following delivery. “I just didn’t want to go through anything else right after having the baby. I wanted to go to my six-week check-up and then, you know, and then have the IUD [placed],” said one participant. Others preferred IPP IUD placement as they felt their bodies were more “open” after delivery: “I think that’s the best way just after the birth of a child. Cause you’re pretty much open. Everything is open.”

IPP IUD placement was a favorable alternative to many participants who were ready to be sterilized, but unable to obtain a tubal ligation due to procedural or consent-related restrictions: “It was a last minute thing because I didn’t sign my papers when I was supposed to. I was supposed to get my tubes tied.” Another participant chose an IPP IUD because she did not want to wait six weeks to have a tubal ligation: “He asked me after I gave birth if I wanted to get an IUD now... or do I want to wait six weeks or how long it takes to have my tubes tied. I said, ‘Eh... I’ll just have the [IPP] IUD.’”

### Reflections

Participants overall described congruence of what was explained to them in the office and the events following IUD insertion. “He walked me through every step… He told me exactly what I was going to feel. And it went perfect!” However, of those whose expectations were not met, IUD expulsion and pain were the most common incongruences. “I feel like maybe if they woulda did it after the fact, that the swelling went down, maybe it would have been better,” said a participant whose IPP IUD expulsed. Another participant was pleasantly surprised about how painless her interval IUD insertion was: “I thought it was going to be more uncomfortable.”

Most participants in both groups expressed satisfaction with their choice of insertion timing. “So the first time it was really uncomfortable. But this one I feel like ok, it’s the right decision,” said one participant who previously received an in-office IUD and chose IPP insertion for her next IUD. Even participants with IUD expulsions described an overall positive experience: “I still feel good. I can’t say anything about it even though it came out.”

A few participants with IPP IUDs were distressed by unexpected complications: “I was supposed to come back in and get [an interval IUD]. But, I didn’t like the fact that [the IPP IUD] came out. So, I just went and got a Depo [Provera] shot... I don’t know if I’m gonna get [another IUD]... I’m like scared to get it,” said one participant whose IPP IUD expulsed. Another participant with an unexpected expulsion had a similar reaction: “I would not be interested in getting it again. That’s for sure. I just think like that one month of pain just turned me completely around on it.”

## Discussion

Our study suggests that timing of LNG IUD insertion was influenced by prior experience and knowledge as well as delivery room considerations. For participants who received an IPP IUD, convenience, desire for immediate contraception, pain control and availability of alternative contraception options were most influential. Patients who received an interval IUD performed more outside research, focused on the events surrounding delivery, and favored additional recovery time before obtaining an IUD. Early, frequent, and thorough counseling was viewed favorably when compared to counseling on Labor & Delivery. While overall there was congruence of participant expectations and experiences, expulsion of IPP IUDs was influential in future IUD decision-making for participants whose IUDs expulsed.

Contraceptive counseling is recommended as a routine part of prenatal care [9]. Standardized counseling increases patient satisfaction and insertion of postpartum LARC [10, 11]. In care models with fragmented provider continuity, standardization is especially important. Counseling that includes immediate contraception options is critical during the current COVID-19 pandemic in order to reduce the number of exposures and conserve personal protective equipment [12]. In our study, counseling about insertion timing was heterogeneous and resulted in adverse experiences for some participants. Several participants stated they would have chosen alternative timing if they had been aware of all their options. Lack of knowledge about the availability of alternative placement timing and late counseling led to ignorance, coercion, or fear concerning future IUD use.

Patients should not feel coerced by timing or omission of information. Counseling solely at the time of labor or while being transferred to the operating room should be minimized, and in-office counseling emphasized whenever possible. Counseling should be universal and not based on patient insurance or demographic factors, comprehensive in terms of discussing all contraceptive options, and include plans for LARC removal if desired. These are crucial in maintaining reproductive autonomy and justice [13–15]. Our recommendations for antenatal contraception counseling appear in Table 4.

**Table 4:** Recommendations for antenatal counseling

1. When possible, initiate contraception counseling early in the antenatal period and avoid counseling solely at the time of delivery
2. Ensuring counseling is comprehensive of all options and standardized regardless of patient insurance and demographics
3. Review the patient’s previous contraception experiences including previous IUD use
4. Assess specific goals of contraception initiation timing (i.e. leaving the hospital with effective contraception or adequate recovery time)
5. Discuss both interval and postplacental options with all patients who desire postpartum IUDs as patient values may not be readily apparent
6. Inquire about the importance of anesthesia during IUD insertion
7. Review differing complication rates between postplacental and interval IUD insertion to mitigate expectations

There were several limitations of this study. The study was conducted in a tertiary care center at a safety-net hospital and results may therefore not be generalizable to the entirety of pregnant patients. Interviews were limited by participant availability and willingness to participate in an extended conversation about contraception. Although interviews occurred only six months after LNG IUD insertion, our study is limited by the inherent recall bias associated with retrospective interviewing. Demographic factors also varied between the two groups and statistical analysis of differences between the two groups was not performed given the small sample size and obvious differences. Additionally, only LNG IUD was offered as an immediate LARC option at our institution which may have had an effect on decision-making when compared to institutions with access to more comprehensive immediate contraception options. Data presented in this study are also from before the COVID-19 pandemic and therefore may not be reflective of current practice changes. However, the insights suggested by this study regarding insertion timing decision-making could set the stage for future empiric research analyzing differences in patients who receive IPP versus interval IUD placement. Future studies could specifically explore differences in decision-making between patients who received in-office counseling and counseling on arrival to the labor unit.

## Conclusion

Providers should be mindful that a continuum of factors influence LNG IUD insertion timing. Shared decision-making that takes into account patient beliefs and values should be incorporated into each contraception discussion. Thorough contraceptive counseling should include the availability of IPP and interval IUD insertion options and the distinct risks of expulsion. Counseling about IUD insertion timing should primarily occur antenatally during in-office prenatal care.

## Data Availability

The datasets used and/or analyzed during the current study are available from the corresponding author on reasonable request.

## Abbreviations

LARC: Long-acting reversible contraception
IUD: Intrauterine device
IPP IUD: Immediate postpartum intrauterine device
